# Supplementary material for: CharMark: character-level Markov modeling for interpretable linguistic biomarkers of cognitive decline
Source: Front Digit Health. 2025 Nov 19;7:1659366. doi: 10.3389/fdgth.2025.1659366 (PMC12672863; doi:10.3389/fdgth.2025.1659366)
Supplement: Supplementary file 1 [file Datasheet1.pdf]

Supplementary Listing: Minimal Reproducibility Script

The following Python script demonstrates the preprocessing and feature extraction pipeline used in the study. It reproduces the construction of CharMark features from transcripts and illustrates model validation on a 5-fold cross-validation split.

```python
import string, numpy as np
from sklearn.model_selection import StratifiedKFold
from sklearn.preprocessing import StandardScaler
from sklearn.linear_model import LogisticRegression

# --- Preprocessing ---
def clean_text(text):
    text = str(text).lower()
    allowed = set(string.ascii_lowercase + " ")
    return "".join(ch for ch in text if ch in allowed)

# --- Markov Feature Extraction ---
VOCAB = list("abcdefghijklmnopqrstuvwxyz ")
IDX = {ch:i for i,ch in enumerate(VOCAB)}
V = len(VOCAB)

def transition_matrix(text, alpha=0.01):
    M = np.zeros((V, V))
    for a, b in zip(text[:-1], text[1:]):
        if a in IDX and b in IDX:
            M[IDX[a], IDX[b]] += 1
    M += alpha
    M /= M.sum(axis=1, keepdims=True, where=M.sum(axis=1, keepdims=True)!=0)
    return M

def steady_state(M, iters=500):
    p = np.ones(V) / V
    for _ in range(iters):
        p = p @ M
    return p

def charmark_features(texts, alpha=0.01):
    return np.array([steady_state(transition_matrix(t, alpha)) for t in texts])

# --- Example CV pipeline ---
texts = ["sample transcript here"]   # replace with dataset
labels = [0]                         # 0=AD, 1=HC
X = charmark_features([clean_text(t) for t in texts])

cv = StratifiedKFold(n_splits=5, shuffle=True, random_state=42)
for train, test in cv.split(X, labels):
    scaler = StandardScaler()
    Xtr, Xte = scaler.fit_transform(X[train]), scaler.transform(X[test])
    clf = LogisticRegression(penalty="l1", solver="liblinear",
                             class_weight="balanced", max_iter=1000)
    clf.fit(Xtr, np.array(labels)[train])
    # predictions = clf.predict(Xte)
```
