# Supplementary figures and images for: CharMark: character-level Markov modeling for interpretable linguistic biomarkers of cognitive decline
Source: Front Digit Health. 2025 Nov 19;7:1659366. doi: 10.3389/fdgth.2025.1659366 (PMC12672863; doi:10.3389/fdgth.2025.1659366)

**Aggregated Confusion Matrix — CharMark ( $\alpha = 0.1$ ), AD-positive**

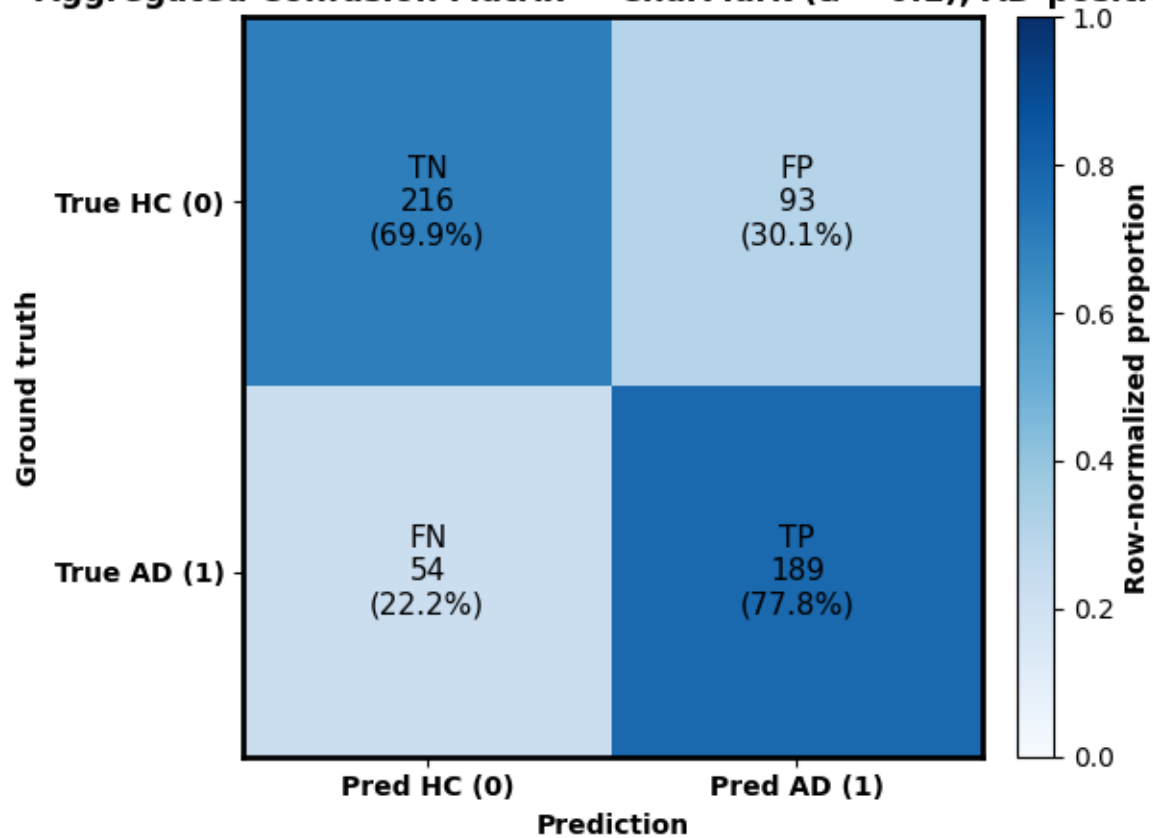

Supplement: Supplementary file 2 [file Image1.pdf]
